# 

**Published:** 1917-06

**Authors:** 


Vol. XXXII, JUNE, 1917 No. 12

### TEXAS Medical Journal

